# Supramolecular Biomaterials for Cancer Immunotherapy

**DOI:** 10.34133/research.0211

**Published:** 2023-09-12

**Authors:** Huan Liang, Qingqing Lu, Jie Yang, Guocan Yu

**Affiliations:** ^1^ College of Science, Nanjing Forestry University, Nanjing 210037, P. R. China.; ^2^ Key Laboratory of Bioorganic Phosphorus Chemistry & Chemical Biology, Department of Chemistry, Tsinghua University, Beijing 100084, P. R. China.

## Abstract

Cancer immunotherapy has achieved tremendous successful clinical results and obtained historic victories in tumor treatments. However, great limitations associated with feeble immune responses and serious adverse effects still cannot be neglected due to the complicated multifactorial etiology and pathologic microenvironment in tumors. The rapid development of nanomedical science and material science has facilitated the advanced progress of engineering biomaterials to tackle critical issues. The supramolecular biomaterials with flexible and modular structures have exhibited unparalleled advantages of high cargo-loading efficiency, excellent biocompatibility, and diversiform immunomodulatory activity, thereby providing a powerful weapon for cancer immunotherapy. In past decades, supramolecular biomaterials were extensively explored as versatile delivery platforms for immunotherapeutic agents or designed to interact with the key moleculars in immune system in a precise and controllable manner. In this review, we focused on the crucial role of supramolecular biomaterials in the modulation of pivotal steps during tumor immunotherapy, including antigen delivery and presentation, T lymphocyte activation, tumor-associated macrophage elimination and repolarization, and myeloid-derived suppressor cell depletion. Based on extensive research, we explored the current limitations and development prospects of supramolecular biomaterials in cancer immunotherapy.

## Introduction

Cancer is one of the most devastating diseases, with continuously increasing new cases affecting millions of people worldwide [1–4]. The American Cancer Society publishes its annual report on cancer statistics, which shows that the overall fatality rate from cancer has diminished by 33% since 1991, with an estimated 3.8 million deaths averted attributed to the deeper comprehension of oncology, which promotes the high-level advancement of tumor diagnosis and antitumor treatment [5–8]. Despite huge progress achieved in drug discovery, cancer is still reckoned as a thorny problem as the leading cause of death [9]. The complicated multifactorial etiology and pathologic microenvironment of tumors caused great troubles in the treatment of disease.

The immune system plays a crucial role in the occurrence, development, and progression of cancer, which should be responsible for monitoring and eliminating abnormal cells, such as cancer cells [10,11]. However, tumor cells can evade the supervision of the immune system through a number of mechanisms, including impairment of immune cell response, up-regulation of immune checkpoint molecules, attenuation of antigen presentation, and induction of immunosuppressive cell differentiation [12–16]. Tumor immunotherapy is a historic landmark in tumor treatment, aiming to recognize and eliminate tumor cells by harnessing the power of intrinsic and adaptive immune systems [17–19]. The activation of adaptive immune response is crucial for predominantly cyto-immune killing [20], with sequential steps as the antigens are released from tumors and recognized by antigen-presenting cells (APCs) in the immune system and form the major histocompatibility complex (MHC) [21,22]. Matured APC further presents the antigen peptide to T lymphocytes and generates antigen-specific signals to prim the cellular immunity dominated by cytotoxic T lymphocytes (CTL) [23,24]. Activated CTL traffics and infiltrates into tumor tissues to recognize and kill cancer cells by releasing granzyme and perforin, and ultimately inhibit tumor growth, invasion, and recurrence [25,26]. In recent years, many immunotherapy methods have achieved promising clinical therapeutic effects, such as tumor vaccines [27,28], immune checkpoint blockade (ICB) [29,30], immunoregulatory therapy [31,32], adoptive CTL therapy [33], and cytokine therapy [34]. Despite certain scenarios success have been achieved, the immune therapy efficacy is thwarted by inadequate immunogenicity of antigen, the unendurable activity of immune cells, insufficient immune response, and disturbance of immunosuppressive cells [35–37]. In addition, there might be some unavoidable side effects of current immunotherapy modalities, which severely limit their clinical applications [38,39]. The rapid evolution of nanomedical science and material science promotes the advanced development in the field of immunotherapy to tackle the above critical issues in past decades [40–42].

**Fig. 1.**
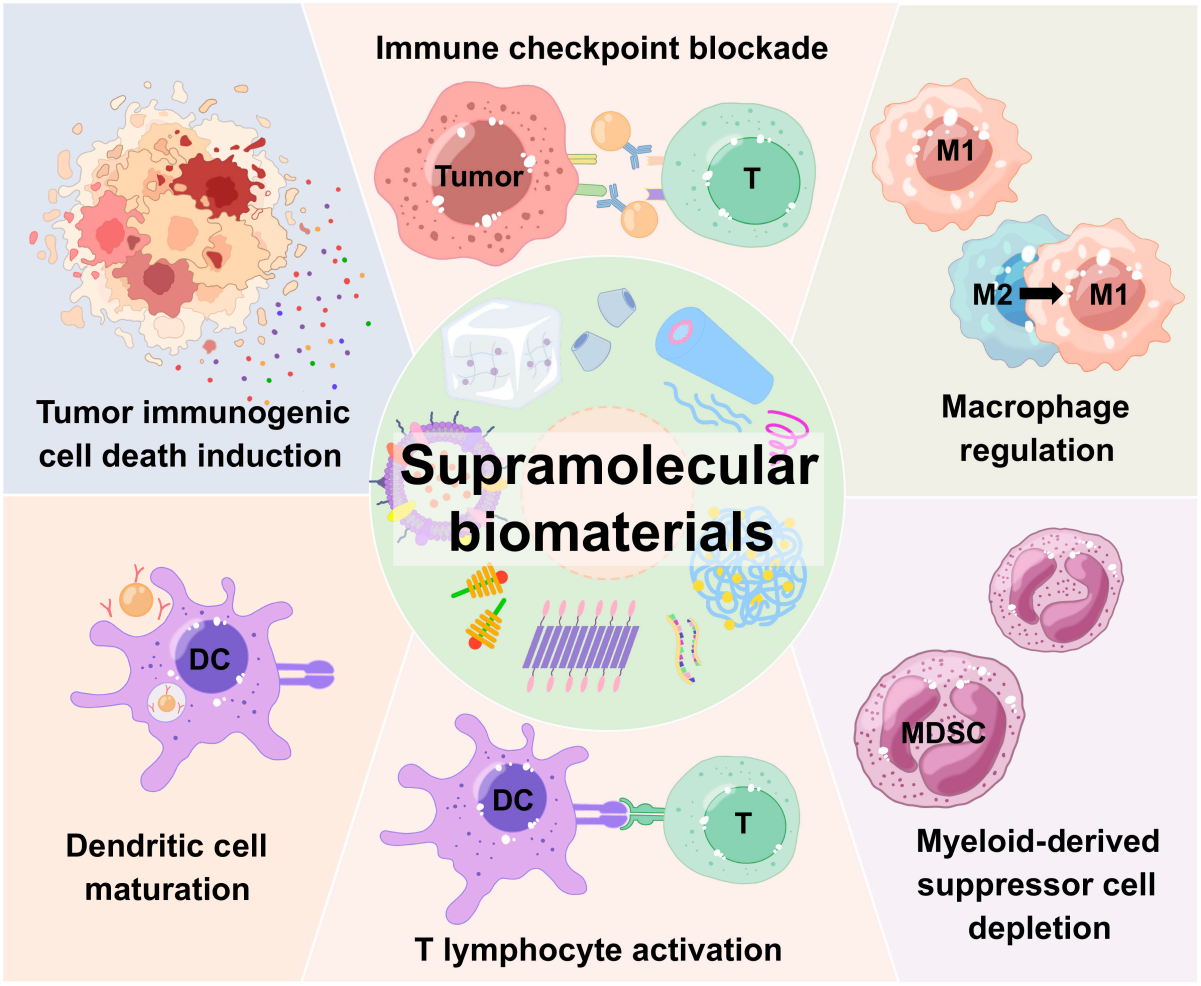
Schematic illustration of supramolecular biomaterials in the modulation of pivotal steps during tumor immunotherapy.

Of these, supramolecular chemistry offers a promising tool of study in the development of immunotherapies for cancer treatment. Supramolecular assembly is that “bottom-up” molecular arrange into well-ordered nanoscale architectures, which was driven by noncovalent interactions, including hydrogen bonding, hydrophobic interactions, π–π stacking, electrostatic interactions, and Van der Waals forces [43–45]. Compared with other nanomaterials, supramolecular biomaterials have emergent properties to develop versatile, flexible, and facile nanoplatforms to prevent tumor growth and immune evasion by fabricating intelligent drug delivery systems or targeting key molecular interactions in immunotherapy [46–48]. In the context of developing novel delivery platforms for immunotherapeutic agents, such as immune checkpoint inhibitors, tumor antibodies, immune activated adjuvants, and cytokines, the supramolecular nanoplatform exhibits unique advantages of high cargo-loading efficiency, excellent biocompatibility, and diversiform immunomodulatory activity due to the modularity assembly of the supramolecular structure [49–51]. Surprisingly, supramolecular biomaterials can be applied to stimulate immune response and regulate immunosuppressive microenvironment homeostasis by modulating their structural properties with suitable shape, size, and surface patterns [52,53]. Collectively, supramolecular biomaterials provide a versatile platform for the development of immunotherapies by enabling the design of molecules and materials that can interact with the immune system in a precise and controlled manner. In this review, we focused on current supramolecular biomaterial systems for immunotherapeutic strategies (Fig. 1) and summarized their crucial roles in the modulation of pivotal steps during tumor immunotherapy, including antigen delivery and presentation, T lymphocyte activation, tumor-associated macrophage elimination and repolarization, and myeloid-derived suppressor cell depletion. Furthermore, the current limitations and development prospects of supramolecular biomaterials in cancer immunotherapy were also explored. Based on extensive research, our review will open a promising avenue for researchers in supramolecular biomaterial development and also propose potential research directions for tumor immunotherapy.

## Application of Supramolecular Biomaterials in Boosting Immunogenic Cell Death-Induced Immunotherapy

As a representative immunotherapy strategy, immunogenic cell death (ICD) could kill the solid tumors in situ, as well as elicit the immune response to realize the elimination of tumor cells to the maximum extent [54]. ICD is a distinctive pattern of cell demise, which achieves activation of antitumor immune responses by releasing tumor-associated antigens (TAAs) [55] and damage-associated molecular patterns (DAMPs) [56]. Taking the advantages of multifunctionality and tunability, the application of supramolecular biomaterials for boosting ICD-induced tumor immunotherapy has been greatly explored. Recent works on bioactive supramolecular nanomaterials as delivery platforms and ICD inducers are presented in this section.

Drug-induced ICD is currently the holy grail to achieve both cytotoxicity and immune elicitation [57]. However, the efficacy of conventional ICD inducers is often limited by short circulation time and poor tumor targeting ability [58]. Thus, the desired immune activation effect cannot be obtained at low dosage, and high-dosage chemotherapeutic drugs would lead to leukopenia [59]. Supramolecular biomaterials could serve as a nanoplatform to improve the therapeutic efficiency of ICD inducers with reduced side effects. Zheng et al. [60] proposed a doxorubicin-loaded mesoporous silica nanoparticles (DOX@MSN). In this system, β-cyclodextrin (β-CD) was threaded through a benzimidazole–polyethylene glycol (PEG)–ferricinium rotaxane gate to realize the acidic and redox dual responsive DOX release and amplified the anticancer immune response. Qi et al. [61] reported cell membrane vesicles based on supramolecular technology (SCMVs) to load indocyanine green (ICG) through the host–guest complexation between β-CD and adamantane. The SCMVs could specifically accumulate in tumors, mediating ICD through photodynamic therapy. This type of supramolecular engineering cell membrane vesicles presents a friendly and generalizable strategy for precise tumor immune therapy.

**Fig. 2.**
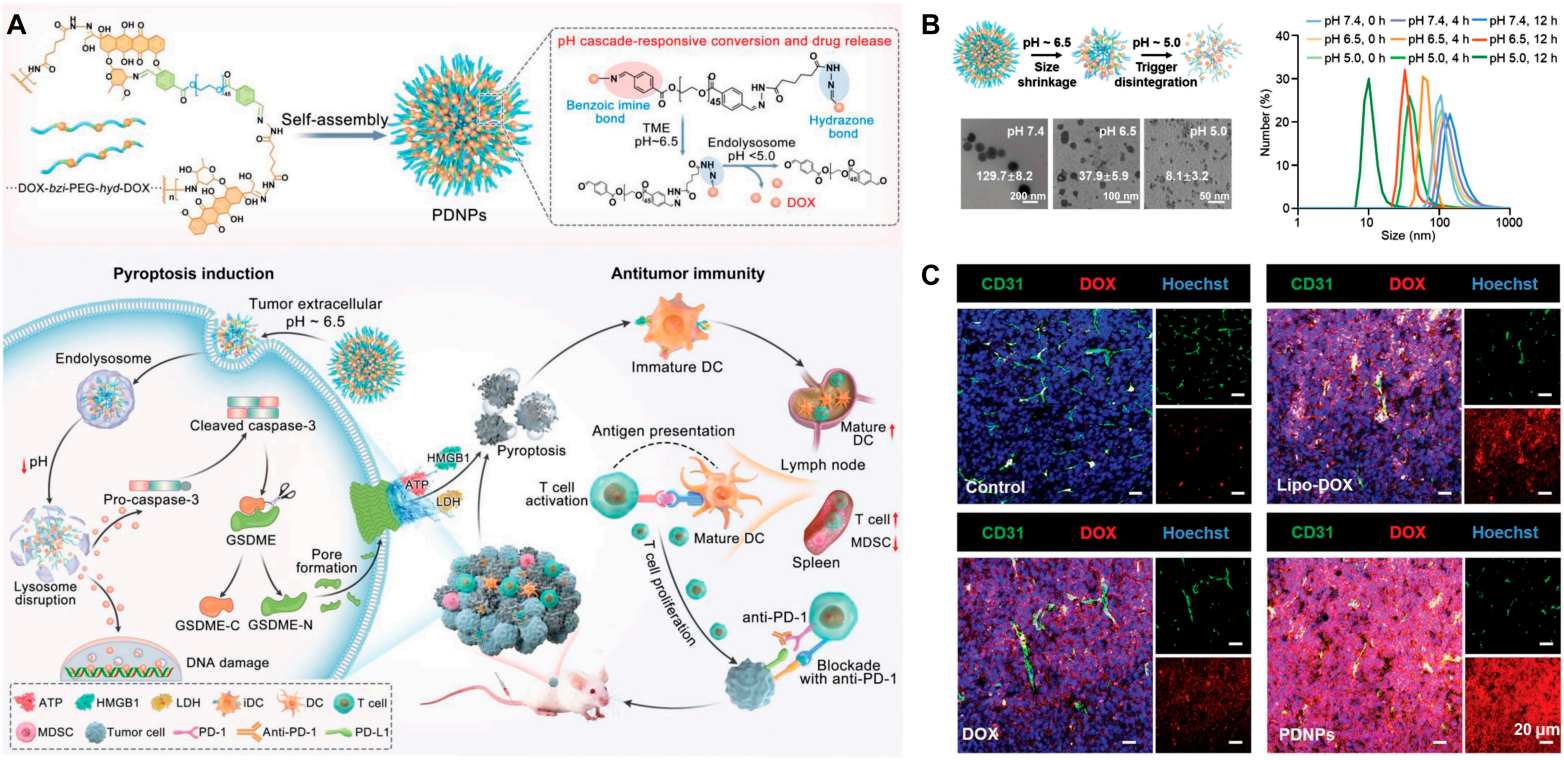
(A) Schematic illustration of size-transformable supramolecular nanoprodrug (PDNP)-mediated cancer chemo-immunotherapy. (B) pH-triggered sequential conversion of particle size over time. (C) Representative immunofluorescence staining of excised tumor tissues after PDNP treatment for penetration studies. Reproduced with permission from [67]. Copyright 2022 Wiley-VCH GmbH.

In many cases, the therapeutic outcomes of nanomedicines show unsatisfactory improvement due to the complicated delivery journeys [62]. Different requirements are proposed to satisfy the drugs for acting at different sites. Theoretically, larger nanoparticles with a size of around 100 nm tend to accumulate at tumor sites under enhanced permeability and retention (EPR) effect [63]. However, the malformed vessel and dense extracellular matrix of solid tumor tissues cause an interstitial hypertension microenvironment, hampering the deep penetration of nanoparticles [64]. Therefore, the nanomedicines with smaller particle size (<30 nm) are required to alleviate diffusional resistance and potentiate intratumor penetration [65]. It requires even much smaller size to cross the nuclear pore (<9 nm) [66]. The supramolecular biomaterials could be applied to responsively modulate their structural properties with suitable shape, size, and surface patterns. Xu and colleagues [67] proposed drug–polymer supramolecular nanoparticles (PDNPs) as an ICD inducer to boost the immunogenicity of tumor (Fig. 2). PDNPs contain 2 different acid-sensitive cleavable linkers, which could display a graded response to the increasing acidity of the tumor microenvironment (TME). At physiological conditions, the PDNPs exhibited a spherical shape and remained stable with a size of around 129.7 ± 8.2 nm, ensuring adequate tumor accumulation. The PEG shielding begins to detach from PDNPs, owing to the protonation of the benzoic imine bond at acidic TME (pH ≈ 6.5). In this process, the nanoparticles shrink to 37.9 ± 8.2 nm, promoting the deep tumor penetration of nanoprodrugs. After endocytosis, second-stage shrinkage of PDNPs occurred due to the cleavage of acidic-sensitive hydrazone bond at endolysosome environment (pH ≈ 5.0), leading to complete decomposition into 8.1 ± 3.2 nm. Multi-stage size regulation strategy promotes the accumulation, retention, and penetration of PDNPs and lays the foundation for the accurate release of drugs at target sites. Finally, the PDNPs consummately provoke pyroptosis and facilitate ICD procedure, thereby boosting the antitumor immune response.

**Fig. 3.**
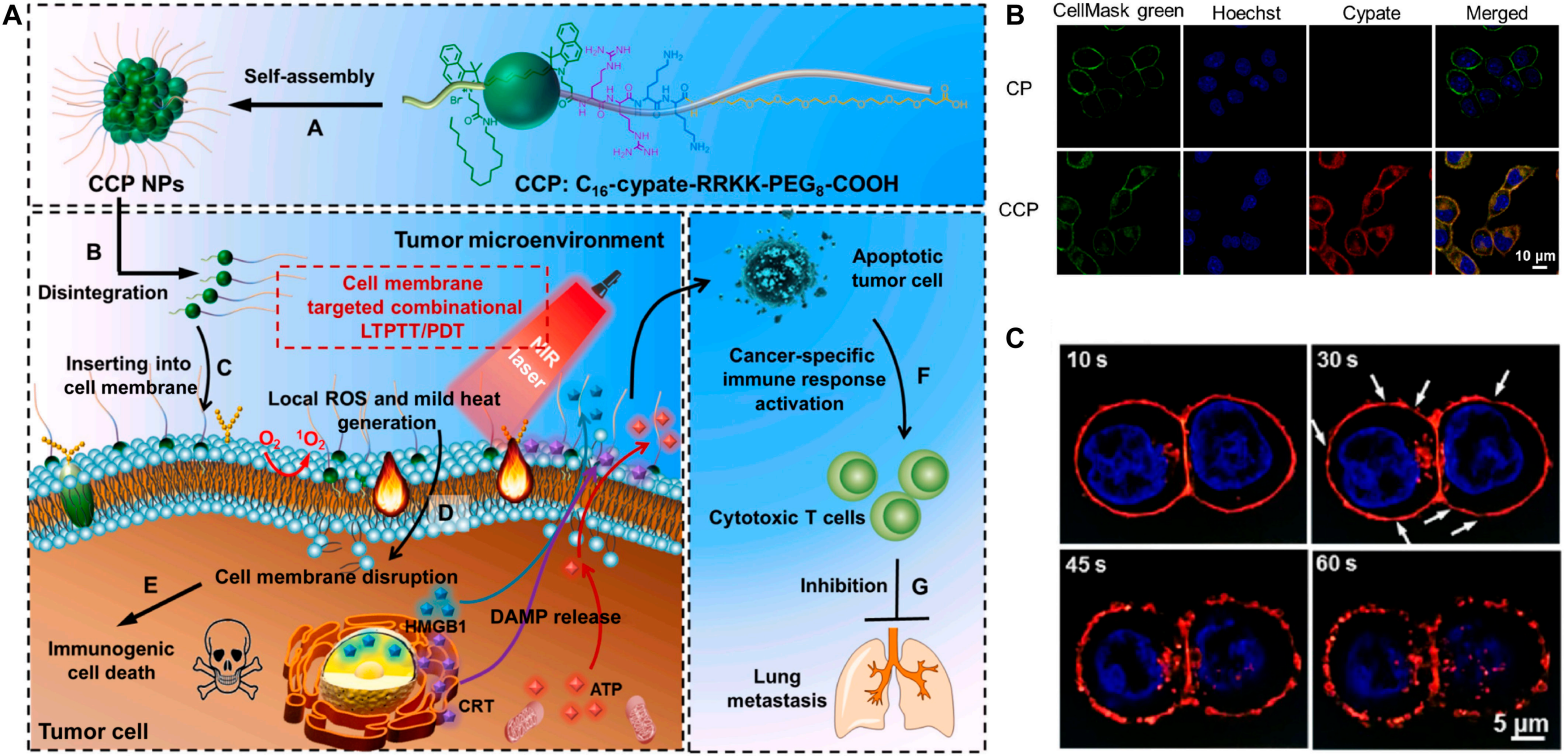
(A) Schematic illustration of cell membrane-targeting self-delivery nanodrugs for high-efficient and anti-metastatic combinational LTPTT/PDT (low-temperature photothermal therapy/photodynamic therapy). (B) CLSM images of 4T1 cells treated with CP (Cypate-RRKK-PEG8-COOH) and CCP (2 μM) for 30 min. (C) CLSM images of the cell membrane disruption of CCP (2 μM) pretreated 4T1 cells under high power intensity of NIR laser. Reproduced with permission from [69] . Copyright 2022 Elsevier Ltd.

In addition, the specific supramolecular biomaterial could induce direct targeting and disrupt tumor cell membranes, leading to quick destruction of the intracellular homeostasis, without the requirement of traditional drug delivery systems to overcome intracellular delivery barriers [68]. Zhang and colleagues [69] reported a tumor cell membrane targeted chimeric peptide [C_16_-cypate-RRKK-PEG_8_-COOH (CCP)]. The CCP could assemble to form a self-delivery system under noncovalent forces. Due to the positively charged moiety displaying a high affinity to the negative charged membrane by electrostatic interaction, the RRKK peptide could insert into the cell membrane (Fig. 3). Subsequently, the self-assembled CCPs could be localized to generate reactive oxygen species (ROS) and mild heat (<45 °C) under near-infrared (NIR) light irradiation, which directly wipes out the tumor cell membrane and induce ICD to achieve immunotherapy. iRGD as a cell penetrating peptide could specifically recognize tumor vascular endothelial cells by binding to the excessively expressed integrin receptors, thus facilitating the deep penetration of drugs into tumor tissues [70]. Therefore, iRGD peptide modification could exhibit a high-performance delivery strategy for ICD inducers to effectively initiate the immune response [71].

The peptide with surface-induced assembly property could trigger the formation of supramolecular nanoclusters on cell membranes, thus leading to enhanced endocytosis and organelle distribution and retention [72–74]. According to a previous report, the lysosomal accumulation of nanoparticles and ROS generation could cause lysosomal membrane permeabilization and induce ICD [75]. Based on that, Ding and colleagues [76] designed TPA-FFG-LA, which could bind to the cell surface through high affinity with epidermal growth factor receptor (EGFR). Subsequently, the hydrophobic amino acid sequence of TPA-FFG-LA could assemble on the cell membrane, resulting in the uptake and lysosomal accumulation of nanoclusters. Under laser irradiation, the AIEgen TPA-S-RDN generated lots of ROS and caused cell death. The results imply that TPA-FFG-LA could excellently realize synergetic lysosomal membrane permeabilization and ICD by the strategy of peptide assembly and AIEgen-based photodynamic therapy to eradicate tumor cells.

## Applying as Delivery Platform for ICB Therapy

ICB has shown great promise by harnessing the blockage of inhibitory signals in the immune system to combat tumors [77]. The research on programmed cell death protein 1 (PD-1) and PD-1 ligand (PD-L1) pathway has gained prominence [78] due to their high clinical efficacy. The tumors overexpressed PD-L1 on the cell membrane to evade the PD-1^+^ T cell recognition, resulting in T cell exhaustion and anergy [79,80]. Therefore, blocking the interactions between PD-1 and PD-L1 with antibodies [immune checkpoint blockers (ICBs)] is considered capable of restoring T cell function, leading to the long-term anticancer immune response [81,82]. However, only a relatively little fraction of patients obtained direct benefit from the ICB treatment, exhibiting less than 30% response rate in the clinic [83]. The principal reason is that the infiltration of tumor-specific T cells is deficient in most patients [84]. Furthermore, the off-target binding of ICBs with normal tissues can even cause serious immune-related adverse events [85]. Supramolecular biomaterial-based drug delivery systems are highly appealing strategies to controllable and sustainable release of various bioactive agents for overcoming these limitations in ICB therapy.

The therapeutic dilemma of immune agent stems from the intrinsic or acquired resistance environment within tumors [86]. It is being realized that the co-delivery of therapeutic and immune regulation agents might be the optimal approach to achieve the maximal antitumor efficacy [87,88]. With this appeal in mind, Yang and colleagues [89] reported that a supramolecular “trident” (IND-G^D^F^D^F^D^Y-DPPA-1), consisting of ^D^PPA-1 (as the antagonist against PD-L1), indoximod (as the indoleamine 2,3-dioxygenase inhibitor), and D-tetrapeptide of G^D^F^D^F^D^Y (as self-assembly module), displays triple functions for boosting the tumor immunotherapy. Besides, the phosphatidylinositol 3-kinase (PI3K)-AKT and mitogen-activated protein kinase (MAPK) pathways have been investigated to modulate the expression of PD-L1 in different cancers [90,91]. However, the inhibition of PI3K or MEK might exist potential toxicities existed for modulating T cell functions [92,93]. Supramolecular technology-based nanocarrier development allows the selective delivery of therapeutic agent payload to tumor tissues [94]. Sengupta and colleagues [95] designed PI3K and MEK inhibitors as molecular subunits of quantum mechanical all-atomistic simulation-based supramolecular assembly for PD-1–PD-L1 ligation regulation. The supramolecular nanocarrier could be applied for stable and efficient loading therapeutic drugs into tumors. Increased intratumoral therapeutic agents resulted in a sustained pharmacodynamic effect for enhancing antitumor efficacy. Finally, the combination of supramolecular-based targeted therapy and ICB treatment carries out an enhanced anticancer outcome in breast and melanoma cancer.

**Fig. 4.**
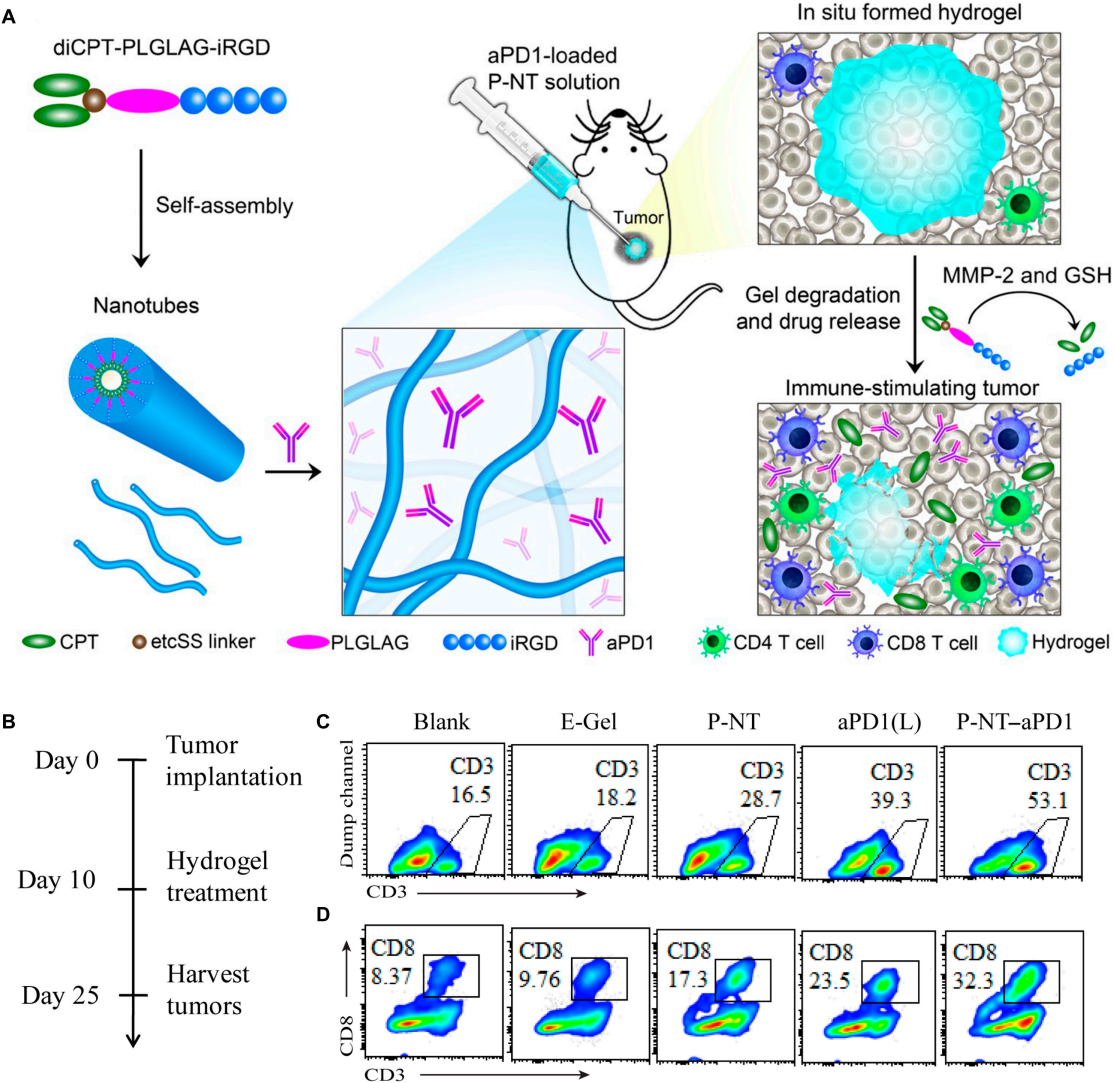
(A) Schematic illustration of localized CPT and anti–PD-1 delivery using an in situ formed supramolecular hydrogel to attain bioresponsive drug release and TME regulation. (B) Experimental schedule. (C) Representative flow cytometric analysis of CD3^+^ T and (D) CD8^+^ T cell infiltration within the tumor by different treatment groups. Reproduced with permission from [98]. Copyright 2020 The Authors, some rights reserved; Exclusive licensee American Association for the Advancement of Science. No claim to original U.S. Government Works. Distributed under a Creative Commons Attribution NonCommercial License 4.0 (CC BY-NC).

Supramolecular hydrogels as “smart” delivery system allows the site-specific delivery and sustained release of checkpoint inhibitors [96]. As a local delivery strategy, supramolecular hydrogels could significantly decrease the adverse events of ICB therapy caused by off-target[97]. Therefore, Wang et al. [98] developed a drug-based supramolecular hydrogel to encapsulate anti–PD-1 antibodies for in situ ICB therapy. In this work, the amphiphilic peptide hydrogelator can assemble into nanofibers, serving as a reservoir for long-term release of camptothecin and anti–PD-1 within the TEM (transmission electron microscope) (Fig. 4). The 2-component system displays a high potency for immune response stimulation and cancer regression. Yang and colleagues [99] explored a thermo-responsive hyaluronic acid-based supramolecular hydrogel (HA-DEG/UPy), which is formed by both hydrophobic interactions and the quadruple hydrogen bonding. HA-DEG/UPy could facilitate the immunogenic phenotype by DOX and amplify the immune response rates via the blockage of PD-L1 by the ^D^PPA-1 peptide. Wang and colleagues [100] reported a supramolecular nanofiber (TAP) fabricated with a hydrophobic AIE agent (tetraphenylethylene) and a PD-L1 targeted peptide. TAP exhibited a specific affinity to PD-L1 and assembled into nanofibers under the noncovalent interactions. Under laser irradiation, TAP enabled thermal ablation of the tumor to generate TAAs and trigger immunological events against cancer. The abovementioned supramolecular hydrogels are highly appealing strategies to achieve enhanced tumor immunotherapy with minimized immune-related side effects.

**Fig. 5.**
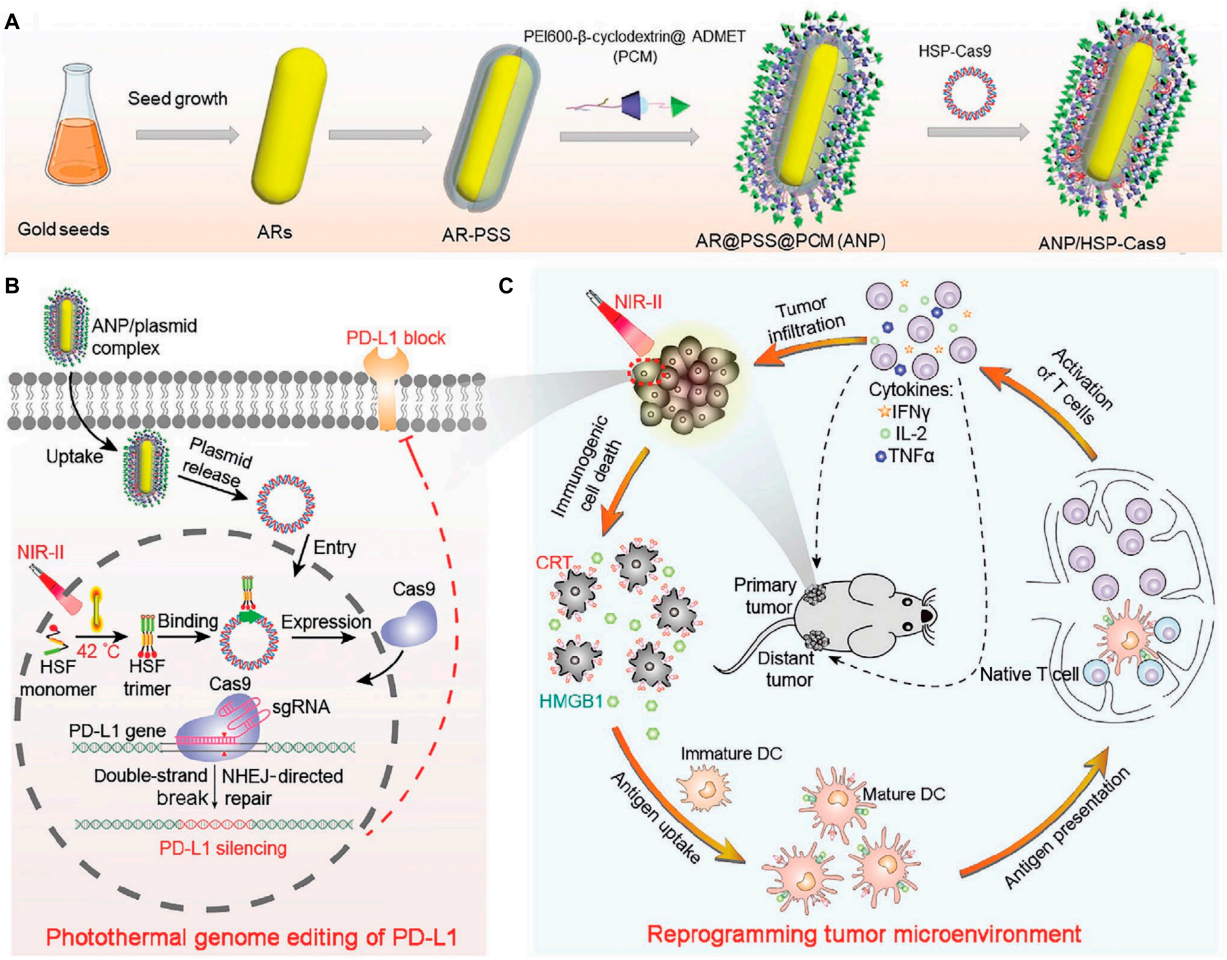
Schematic illustration of the photothermal genome-editing strategy for cancer immunotherapy. (A) Process of preparation of ANP/HSP-Cas9 plasmid complex. (B) Illustration of photothermal activation for PD-L1 genome editing in tumor cells. (C) Photoactivable CRISPR-Cas9 strategy reprograms immunosuppressive tumor environment. Reproduced with permission from [105]. Copyright 2021 Wiley-VCH GmbH.

Moreover, the compensative expression of PD-L1 on the tumor membrane remains continuous even after conformational blockade by anti–PD-L1 antibody [26]. Recently, genome-editing technology has shown great promise as powerful tactics for changing targeted protein genomes from the genomic level [101]. The precise silencing of PD-L1 genome by clustered, regularly interspaced, short palindromic repeats (CRISPR) and CRISPR-associated protein 9 (Cas9) offers a reliable approach for circumventing the dilemmas of traditional ICB therapy [102–104]. Ping and colleagues [105] established supramolecular cationic gold nanorods that simultaneously serve as a carrier to deliver CRISPR/Cas9 for PD-L1 genome-editing and second NIR-window (NIR-II) thermotherapy (ANP/HSP-Cas9) (Fig. 5). The gold nanorod harvested the NIR-II light and generated mild hyperthermia (42 °C) to activate ICD, as well as provided an optimal environment temperature for transcriptional activation of Cas9. The mild hyperthermia can precisely induce the ICD to circumvent the high temperature-related unfavorable inflammatory responses and damage of the healthy tissues [106]. The ICB-enhanced immunotherapy proved the superior ability for killing the remaining tumor cells and inhibiting the metastasis of cancer. Collectively, the synergetic strategy of photothermal therapy and genome-editing technology readily reprograms the TEM to realize ICB-based tumor immunotherapy.

## Using as an Immune Response Enhancer for Dendritic Cell Maturation

Dendritic cells (DCs) are professional APCs, with unique ability to capture, process, and present the antigens in tissues and peripheral blood, considered as critical factors in antitumor immunotherapy [107–109]. In general, immature DCs are skilled in ingesting and converting antigen proteins into peptides. Then, the DCs initiate their migration from peripheral tissues to lymphoid organs, as well as transition to matured APCs by presenting the antigen peptide on the MHC molecules [110,111]. The formation of MHC-peptide complexes, expression of chemokine receptors, regulation of costimulatory molecules, and production of cytokines from DCs are crucial for antigen-specific T cell activation [112–115]. The tumor could create a hostile environment that leads to immune evasion by weakening DC activity or conditioning DCs to form suppressive T cells [29,116]. Therefore, improving the maturation and antigen presentation level of DCs can be marshaled as a promising strategy for the prevention and therapy of tumors. With decades of efforts, many distinctive hallmarks of the DCs are gradually getting clear, which brings opportunities to regulate the cell function and vitality to facilitate anticancer therapeutic efficacy [117–119]. There are several factors that proved to be effective in inducing DC maturation and facilitating the proinflammatory phenotype. It is demonstrated that the components of pathogen-associated molecular patterns from viruses and bacteria, such as CpG, viral nucleic acids, and lipopolysaccharides, could promote DC maturation through Toll-like receptor (TLR) pathway [120–122]. The environmental cytokines, including interleukin-10 (IL-10) and tumor necrosis factor-β (TNF-β), also act as important contributors to the regulation of DC phenotype [123,124]. Based on previous research, the DC-mediated therapeutic approaches are currently presented.

One promising therapeutic strategy is ex vivo generating DCs loaded with tumor antigens and re-injected back to patients, thereby inducing the antigen-specific T cells against cancer [125]. After intradermal administration, the matured DCs tend to migrate and recognize the T cells to induce strong immunity [126]. In general, the DCs represented an inefficient homing capability, with less than 5% of intradermally injected DCs targeting draining lymph nodes [127]. Direct injection of matured DCs into lymph nodes could circumvent the skin migration issues, achieving the enhanced efficacy of immune response [127,128].

**Fig. 6.**
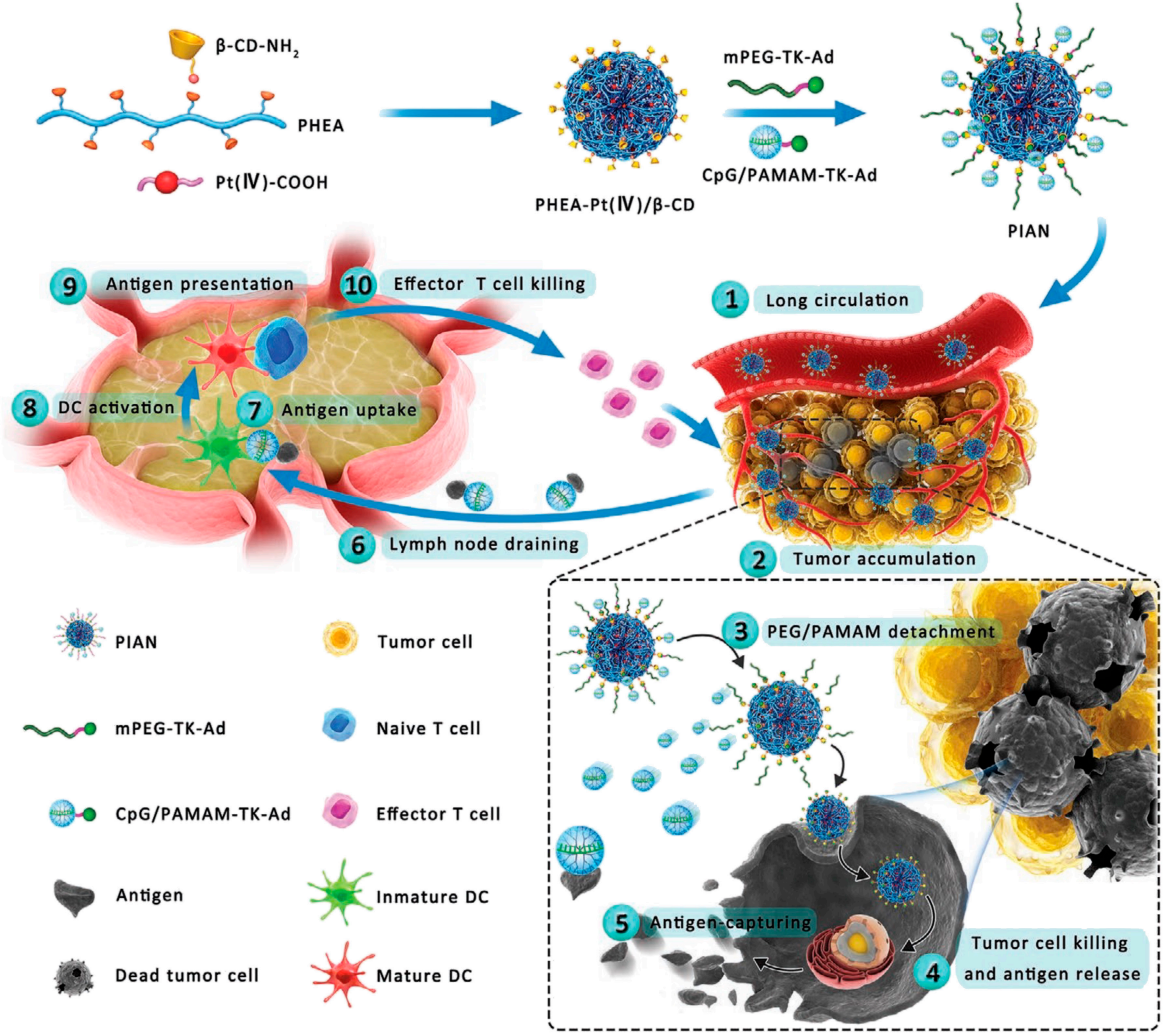
Schematic preparation of the programmable immune activation nanomedicine (PIAN) for immune activation and tumor inhibition. PIAN is fabricated through a one-step supramolecular assembly process via β-CD/adamantine (Ad) host–guest interactions among various components. Reproduced with permission from [46]. Copyright 2021 Wiley-VCH GmbH.

Another DC-based therapeutic strategy in tumor treatment is mobilizing DCs directly in vivo, usually targeting the DCs within tumors or lymphoid organs. Vaccines have been widely investigated against tumors by modulating the body’s immune activity [129]. Proteins and peptides were considered as suitable antigens to generate cellular or humoral immunological responses [130,131]. However, the poor stability and immunogenicity are major limitation for their clinical transformation [132,133]. Varieties of adjuvants have been proposed to enhance the DC maturation, such as TLR agonist and proinflammatory cytokines engineered proteins [134,135]. Therefore, the antigen and adjuvant are required in vaccine fabrication to efficiently generate immune response [136]. Yang and colleagues [137] found that the hydrophobic short peptides could fabricate supramolecular hydrogels and nanofibers through co-assembly with proteins. Then, the adjuvant potency for vaccine formation of both L- and D-peptide-based supramolecular hydrogels was further evaluated [138]. As indicated in the results, compared with the traditional alum adjuvant, the supramolecular hydrogels fabricated in this work could folds increase the immunoglobulin G (IgG) production rate by ovalbumin (OVA). Li and colleagues [139] showed a well-established DNA supramolecular hydrogel network containing unmethylated cytosine–phosphate–guanine (CpG) single-stranded DNA for TLR9 activation [DNA supramolecular hydrogel vaccine (DSHV)]. The fabricated DSHV system could mimic the physiological function of lymph nodes, providing a place rich in CpG to facilitate DC recruitment and maturation. In short, the supramolecular system could serve as a nanoplatform to generate strong immune response and benefit antitumor therapy. Although nanotechnology has made great strides in accelerating the development of immunotherapy, there are still obstacles and paradoxes in fulfilling all demands for intricate immune activation [114,140–142]. Therefore, a programmable supramolecular nanomedicine assembly for multiple steps of immune activation was reported [46]. Zhang et al. proposed a programmable supramolecular nanomedicine (PIAN), which formed a complicated nanostructure with simple modules under the host–guest interactions between cyclodextrin and adamantane. As demonstrated in Fig. 6, the PIAN consists of poly-[(*N*-2-hydroxyethyl)-aspartamide]-Pt (IV)/β-CD, CpG/polyamidoamine-thioketal-adamantane, and methoxy poly(ethylene glycol)-thioketal-adamantane. After intravenous injection, the PIAN can lead to sequential multistage transformation for antitumor immunotherapy, including remaining stable in circulation and efficiently accumulating in the tumor tissues, as well as detachment of the protective layer of CpG/PAMAM (polyamidoamine) and PEG in response to intratumoral ROS to obtain enhanced cellular endocytosis for tumor killing. Then, CpG/PAMAM captures the antigens released from dying tumor cells and migrate to tumor-draining lymph nodes for exerting antitumor immune response. Collectively, the PIAN could act as a nanovaccine to meet the multifunctional requirement of immune activation and anticancer therapy.

## Applying as Regulator of Macrophages

Tumor-associated macrophages (TAMs) account for approximately half of a tumor mass [143]. There exist 2 distinct subtypes of TAM: pro-inflammatory M1 polarized phenotype and immunosuppressive M2 phenotype [144,145]. Therefore, TAMs could act like a double-edged sword in the modulation of tumor growth, metastasis, and invasion [146–148]. Of which, the M1 TAMs display a marked tumoricidal effect to support prolonged patient survival [149]. On the contrary, the M2 macrophages could induce the tumor invasion by suppressing the cytotoxicity of T cells and causing tumor favorable TEM, especially during the early tumorigenesis process [150,151]. Therefore, the development of biological strategies for eliminating M2 TAMs or repolarization of the M2 phenotype to M1 TAMs to modulate tumor growth has been increasing as the forefront of tumor therapeutic research [152–154]. Encouragingly, the supramolecular-based nanomedicines could be explored to selectively target the TAMs and modulate their properties [155]. Hence, the recent progress achieved in TAM regulation based on supramolecular technology to elicit optimal tumor immunotherapy was summarized in this section.

**Fig. 7.**
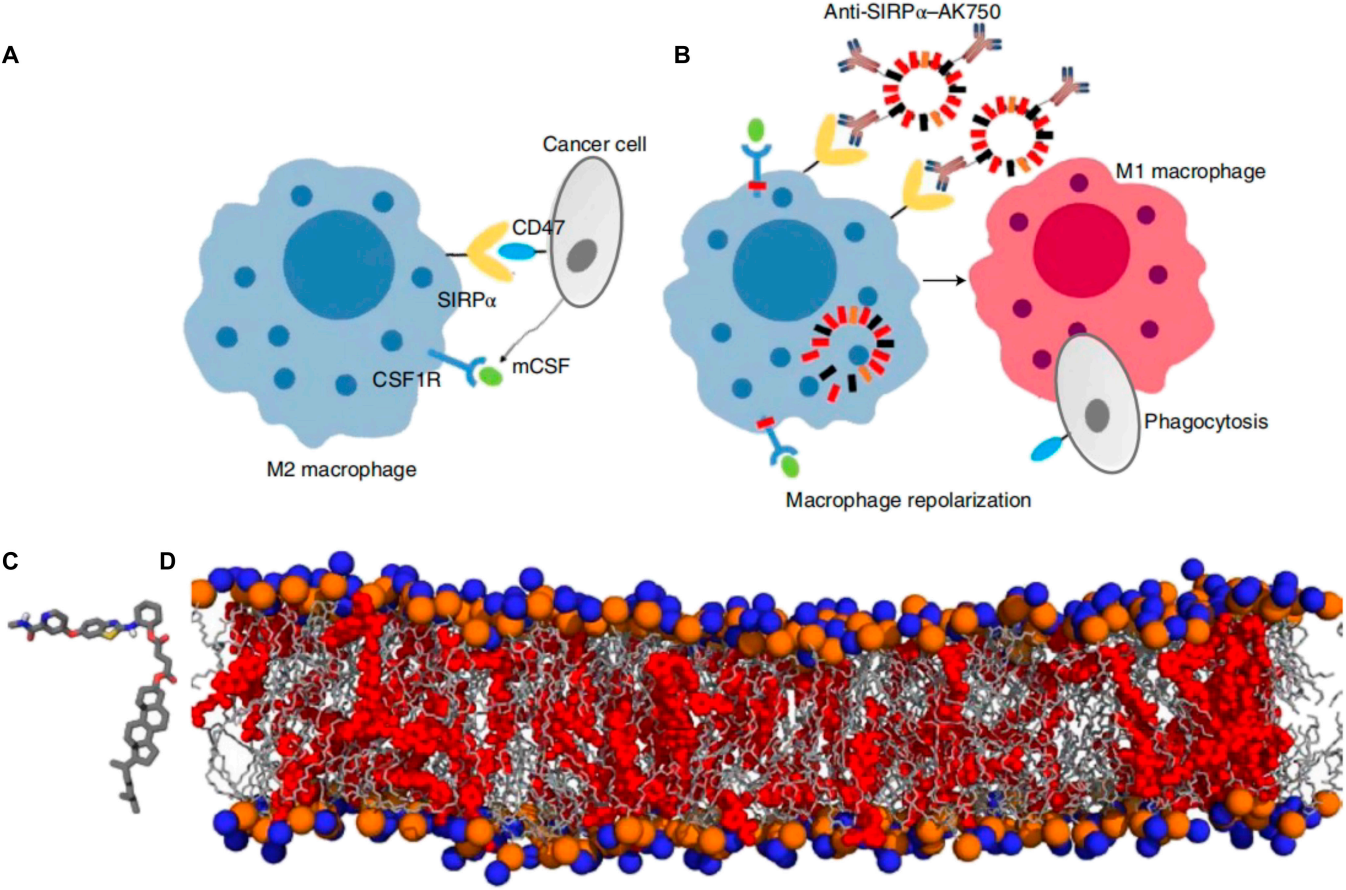
(A) Schematic shows that cancer cells exploit CSF1R signaling to polarize macrophages to the immunosuppressive M2 phenotype and SIRPα–CD47 interactions to inhibit phagocytosis. (B) Schematic illustration of efficient repolarization of an M2 macrophage to the effector M1 phenotype by dual-function supramolecular therapeutic mediated sustained inhibition of CSF1R signaling and enhanced phagocytosis of cancer cells following inhibition of SIRPα. (C) Representation of the quantum mechanical-optimized structure of the molecular subunit of the supramolecular nanostructure. (D) Snapshot of an all-atomistic simulation. Reproduced with permission from [174]. Copyright 2018 Macmillan Publishers Limited, part of Springer Nature.

Recent works have demonstrated positive results of repolarization of TAMs into M1 phenotype to decrease tumor progression [156–158]. Therefore, several kinase inhibitors have been investigated to adjust the cytokines and chemokines of TAMs, which are crucial in macrophage recruitment and differentiation [159–161]. Supramolecular-based nanoparticles are employed as delivery platforms for these TAM-modulated inhibitors and antibodies. The macrophage colony-stimulating factor (MCSF) cytokines are produced by tumor cells with the function to recruit and polarize TAMs into M2 phenotype by binding with colony-stimulating factor 1 receptor (CSF1R) [162,163]. Kinase inhibitors, such as pexidartinib, emactuzumab, and IMC-CS4, were used to target the CSF1R axis in clinical and preclinical trials [164–166]. However, the single therapeutic agent application displayed limited efficacy in CSF1R pathway regulation and even induced serious toxicities [167,168]. Kulkarni and colleagues [169] realized that, in addition to CSF1R, sustained modulation of the downstream pathways is also required to achieve high-performance repolarization of M2. The MAPK pathway has recently been reported to act crucial role in M2 TAM growth and proliferation [170–172]. Therefore, the researchers utilized a supramolecular nanoplatform to rationalize synergistic inhibition of both MAPK and CSF1R pathways. The proposed dual inhibitor-loaded supramolecular nanomedicines could efficiently repolarize M2 TAMs to M1 phenotype, thus carrying out a superior antitumor efficacy. Besides, supramolecular nanomaterials assembled from modular bifunctional therapeutics have been reported, which could block both SIRPα-CD47 and MCSF-CSF1R [173]. Such bifunctional supramolecular nanoplatform could be utilized to bind with M2 macrophages and sustained shutdown of the CSF1R signal, leading to a skew of M2 to M1 phenotype (Fig. 7) [174]. In another work, the local immunostimulatory supramolecular hydrogel was proposed for in situ delivery (R848) as a potent TLR agonist to enable TAM repolarization [175]. Weissleder’s research group further modified R848 with adamantane by aromatic linkage, which could be readily carried with β-CD nanoparticles, as well as retained the TLR agonist capability. The fabricated R848-Ad@CDNP represented as a hopeful approach to arresting cancer growth with minimal side effects. Besides, the application of in vitro-transcribed mRNA to reprogram M2 TAMs displayed tremendous achievement in the suppression of tumor invasion [176].

**Fig. 8.**
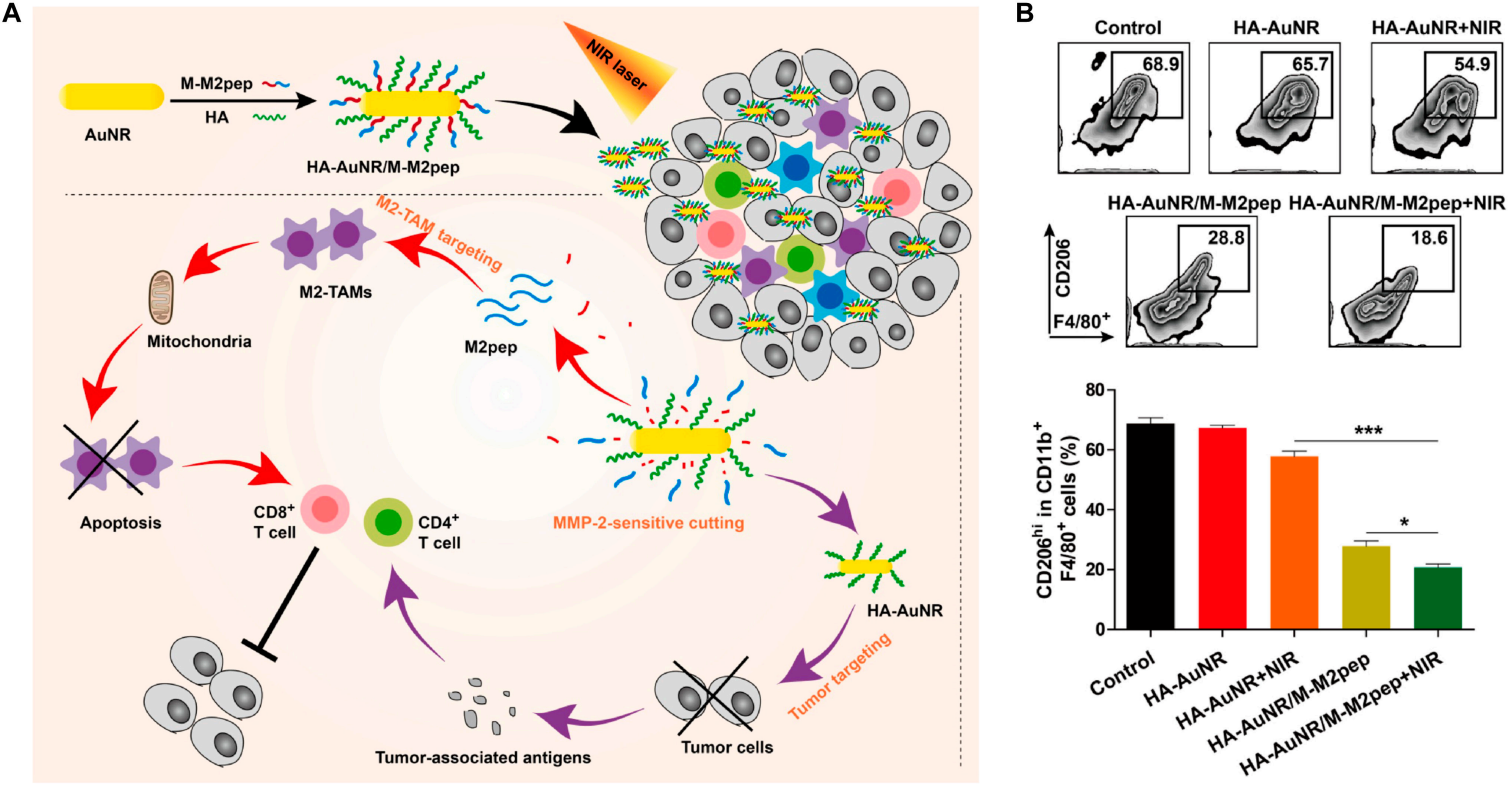
(A) Schematic illustration of enhanced photo-immunotherapy by the combination effect of PTT-induced immune activation and M2-TAM depletion-induced ITME (immunosuppressive tumor microenvironment) modulation based on HA-AuNR/M-M2pep. (B) Representative flow cytometry results and percentages of M2-TAMs in total CD11b^+^ F4/80^+^ cells. Reproduced with permission from [179]. Copyright 2021 Published by Elsevier B.V.

Depletion of M2 phenotype TAMs has been approved as another effective strategy to boost anticancer immune response. However, nontargeted treatments might cause several issues [177,178]. Ai and colleagues [179] designed a targeting nanoparticle (HA-AuNR/M-M2pep NP), which constructing with M2pep fusion peptides (M-M2pep) coated gold nanorods (HA-AuNRs) for specific targeting and depleting M2 TAMs for promoting the photothermal and immunotherapy effect (Fig. 8). In this work, the matrix metalloproteinase-2-responsive M2pep could respond to the tumoral matrix and specifically bind with M2 phenotype TAMs, subsequently consuming M2 to reshape the immunosuppressive TME. Meanwhile, the hyaluronic-mediated efficient endocytosis of AuNR and precise PTT can be achieved under laser irradiation. The synergistic strategy of M2pep-elicited TAM eradication and PTT-based immune activation highlight the great potential to realize the combinatorial anticancer therapeutic in the clinic.

## Application of Supramolecular Biomaterials in Stimulation and Activation of T Lymphocyte

Nanotechnology has concentrated on utilizing the natural capacity of the immune system in eliminating exogenous components to enhance specific immune responses [180,181]. As the most potent immune killers, the cytotoxic T cells have received increasing attention [182]. For instance, chimeric antigen receptor T cell (CAR-T) has been applied to activate T cells to achieve promising tumor immunotherapy [183]. However, the potent and long-term effectiveness of immune response based on this therapy needs to be further improved, due to the limited magnitude of T cell transfusion [184]. Therefore, in situ stimulation and activation of antigen-specific cytotoxic T cells play a critical role in tumor immunotherapy, which has been proven to be a powerful weapon to fight against cancer.

**Fig. 9.**
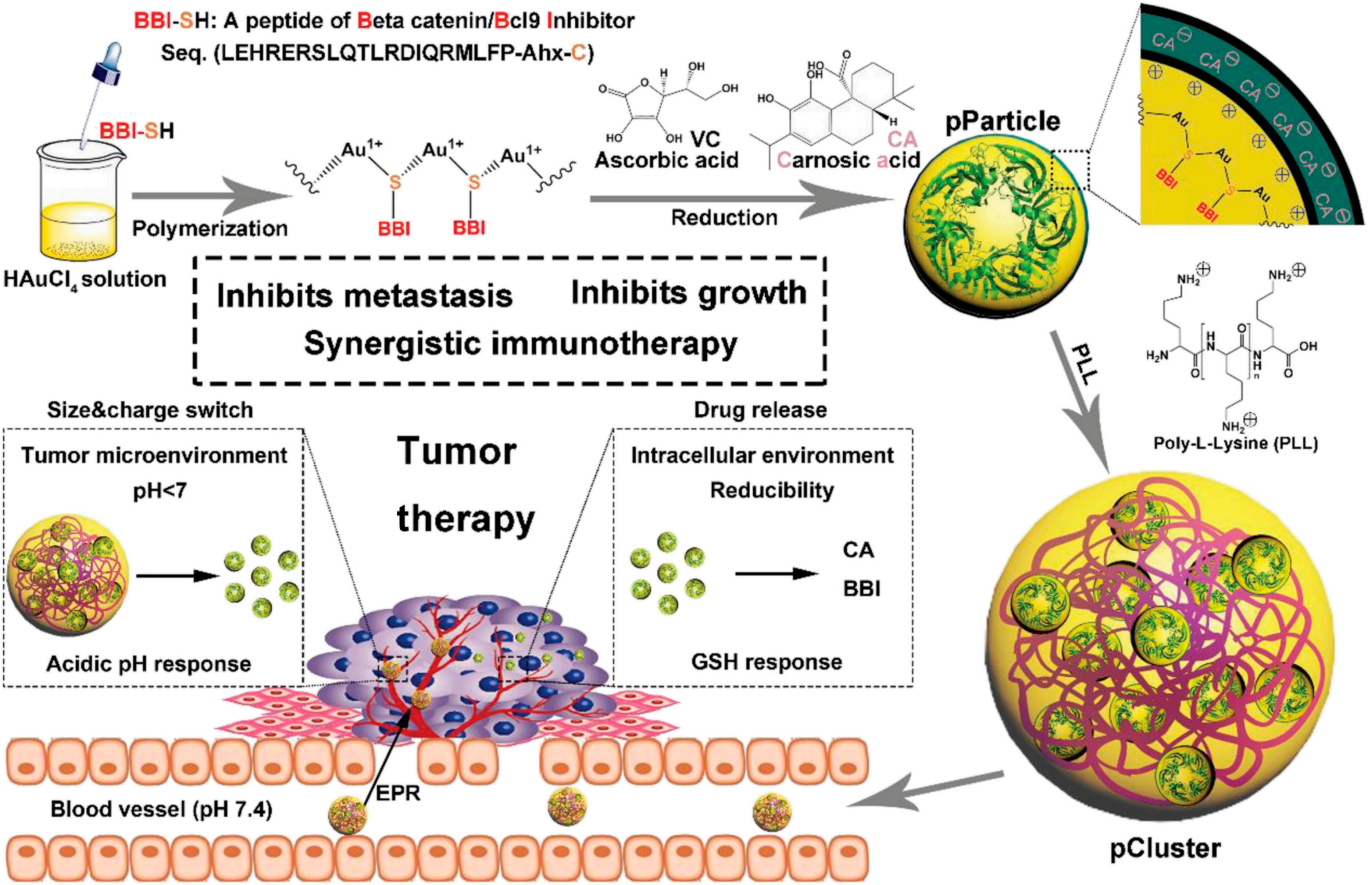
Schematic depiction for synthesis and function of pCluster. Reproduced with permission from [190]. Copyright 2019 WILEY-VCH Verlag GmbH & Co. KGaA, Weinheim.

Autophagy has been reported to contribute greatly to the presentation of tumor antigens and subsequent T cell activation to eliminate tumor cells [185]. Hence, taking a well-designed harness of autophagy might provide a powerful tool in cancer immunotherapy. Wang et al. [186] proposed a supramolecular assembled nanoplatform with the ability to activate the autophagy pathway for facilitating the cross-presentation of antigens and producing specific cytotoxic T cells. It has been mentioned above that PD-1 is overexpressed on the surface of exhausted T cell [187]. The PD-L1 ligand expressed on tumor cells can interact with PD-1 to inhibit the kinase signal and dampen T cell proliferation. Besides, the overexpression of indoleamine 2,3-dioxygenase (IDO) in tumoral tissues also blunts the activity of T cells due to the overdecomposition of tryptophan [188]. Cheng et al. [189] constructed a multifunctional supramolecular nanoparticle for co-delivery of the inhibitor of PD-L1 (^D^PPA-1) and NLG919 as selective inhibitor of IDO to synergistically settle the challenge in tumor immunotherapy. The supramolecular assembly nanoparticle provided a platform for incorporating IDO inhibitor and PD-L1 trap to activate cytotoxic T cells. Subsequently, the drug release behavior of dual tumoral stimulation response ensures the biocompatibility and bioavailability of nanomedicines to facilitate anticancer immunotherapy with minimal adverse effects. Similarly, B cell CLL/lymphoma 9 (Bcl9) is a transcriptional cofactor relative to the tumor progression, which is overexpressed in tumoral tissues with the activity of initiating the Wnt/β-catenin pathway. Toward this mechanism, He et al. engineered a supramolecular nanohybrids with inhibitors of β-catenin/Bcl9 and Au-peptide precursor (Fig. 9). Benefiting from superior biocompatibility, the peptide-mediated nanohybrids have displayed tremendous potential in Wnt/β-catenin signaling inhibition for cancer immunotherapy [190].

The surface properties of self-assembled supramolecular biomaterials are able to modulate immunogenicity. Wen and Collier [191] focus on the research of self-assembling peptide Q11 (QQKFQFQFEQQ), which could allow the formation of supramolecular nanofibers or hydrogels to generate robust antigen-specific immune responses with minimal side effects. It has been confirmed that epitopes within the peptide sequence on T cells are important for supramolecular biomaterial-induced immunogenicity [192]. Peptide Q11 could self-assemble into regular nanofibers without interference from N-terminal properties [193]. Therefore, the influence of surface properties on immunogenicity was further investigated. There were surprising and important findings that the negative surface charge might abolish immune responses against epitope-containing nanofibers. Conversely, the peptide nanofibers with positive surface charge would display enhanced uptake behavior by APCs, maintaining the ability to activate T cell responses.

## Application of Supramolecular Biomaterials in Depletion of Myeloid-Derived Suppressor Cells

High myeloid-derived suppressor cell (MDSC) infiltration is one of the major factors in the formation of immunosuppressive TME to affect tumor immunotherapeutic efficacy [29]. Various drugs have been applied to settle the dysfunction of MDSCs, including 5-fluorouracil, sunitinib, and gemcitabine [194–196]. However, the MDSCs also play positive roles in maintaining physiology homeostasis in normal tissues and organs [197,198]. Accordingly, systemic treatment of chemotherapeutics to suppress MDSCs might induce off-target effects, accompanied by severe side effects. Therefore, nanotechnology-based drug delivery systems have been widely utilized to realize precise depletion of MDSCs in TME with great biocompatibility [199]. The PDE5 inhibitor tadalafil was approved by U.S. Food and Drug Administration to be clinically applied in the treatment of cardiac hypertrophy, pulmonary hypertension, and erectile dysfunction [200]. Fortunately, tadalafil is also reported as a promising candidate to inhibit MDSC activity and restore the immune response function of cytotoxic T cells to facilitate antitumor immunotherapy [201].

**Fig. 10.**
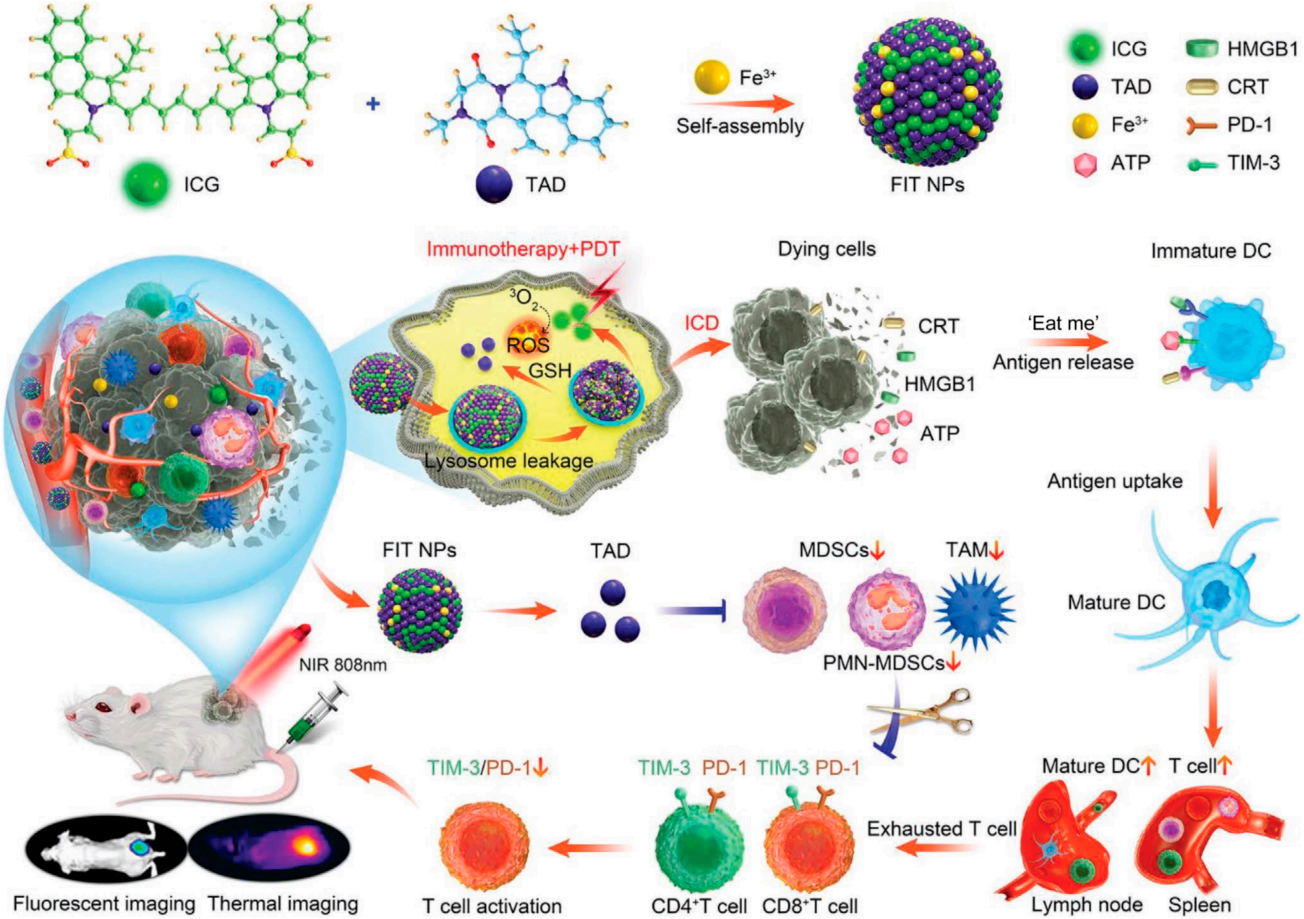
Schematic illustration showing the deductive procedure of FIT nanoparticle for immunotherapy. The preparation process of FIT nanoparticle, and the mechanism of MDSC regulation, ICD induction process, and dual-imaging medicated enhanced cancer immunotherapy. Reproduced with permission from [202]. Copyright 2021 Wiley-VCH GmbH.

Xu and colleagues [202] rationally designed a supramolecular self-assembly system for the co-delivery of tadalafil and ICG (marked as FIT nanoparticles). The obtained supramolecular nanomedicines could improve targeting and delivery efficiency and prolong the blood circulation time of small molecular ICG and tadalafil (Fig. 10). In addition, the photothermal therapy induced by (ICG) was confirmed to generate abundant tumoral antigens, which serve as a personalized tumor vaccine to trigger T cell activation [203,204]. Therefore, the Fe^3+^ coordinated ICG nanoparticles would disintegrate and liberate the therapeutic agents to generate ICD effects and provide an immune stimulation for T cells. Afterward, the tadalafil was released to exhaust MDSCs, as well as reinvigorate cytotoxic T cells. Collectively, the simultaneous immune stimulation and MDSC reduction by co-delivering tadalafil and ICG highlight a superior potential in boosting synchronized cancer immunotherapy.

## Conclusion and Future Perspectives

Supramolecular chemistry-based nanotechnology represents a promising approach for cancer immunotherapy by targeting key molecular interactions involved in tumor growth and immune evasion. This review summarized the recent developments and basic requirements for supramolecular immunotherapy according to the target action of nanomedicines. The supramolecular nanomedicines could be designed to selectively target the tumor antigens, immune checkpoints, TME, and the broader immune system, serving as direct inducers, immune response enhancers, and intelligent delivery systems (Table). (a) Supramolecular agents with therapeutic activities precisely target tumor cells and disrupt tumor cell membranes, thus quickly destructing intracellular homeostasis and killing them. The supramolecular assembly medicines could act as a direct ICD initiator to induce ICD and transfer immune-defective “cold” tumors into immune-activated “hot” tumors, which avoids the requirement of traditional drug delivery systems to overcome intracellular delivery barriers. (b) Supramolecular-based nanoplatforms can be designed to target and stabilize tumoral neoantigens and enhance antigen presentation by promoting the endocytosis and processing of APCs or modulating the expression of MHC molecules on the surface of tumor cells. (c) Supramolecular chemistry-based nanotechnology is a highly appealing strategy to the control and sustainable release of various bioactive agents to block the immune escape of tumor cells by immune checkpoint inhibitor therapy. (d) Supramolecular nanomedicines can also be designed to modulate the activity of the immune system, such as promoting the proliferation and differentiation of cytotoxic T cells, elimination of M2 TAMs, or repolarization of the M2 phenotype into M1 TAM cells, regulating the biofunction of MDSCs, and facilitating the activity of immune effector cytokines. Collectively, the engineered supramolecular nanomedicines could be considered promising candidates for relieving immune resistance and boosting anticancer immunity to realize the tremendous potential merits of tumor immunotherapy.

**Table.** Supramolecular biomaterials for cancer immunotherapy.

Immunotherapeutic strategy	Supramolecular biomaterial	Function	Morphology	Immune agents	Reference
Immunogenic cell death	β-CD/ferricinium	Responsive domain	Nanoparticle	DOX	[57]
	β-CD/adamantane	Carrier	Nanoparticle	ICG/I-MT/resiquimod	[58]
	Drug-polymer	Inducer	Nanoparticle	DOX	[64]
	C_16_-cypate-RRKK-PEG_8_-COOH	Inducer	Nanoparticle	Cypate	[66]
	FFG	Assembly domain	Nanoclusters	TPA-S-RDN	[73]
Immune checkpoint blockade	G^D^F^D^F^D^Y	Assembly domain	Hydrogel	^D^PPA-1/indoximod	[86]
	SOPC	Carrier	Nanoparticle	Selumetinib/supratinib	[92]
	PLGLAG	Assembly domain	Nanotubes/hydrogel	Camptothecin/anti–PD-1	[95]
	HA-DEG/UPy	Carrier	Hydrogel	^D^PPA-1	[96]
	FFVLK	Assembly domain	Nanofiber/hydrogel	Tetraphenylethylene	[97]
	β-CD/adamantane	Carrier	Nanorod	CRISPR/Cas9/Au	[102]
DC maturation	NapGFFpY-OMe	Assembly domain	Hydrogel	OVA	[135]
	DNA network	Carrier	Hydrogel	CpG	[136]
	β-CD/adamantane	Carrier	Nanoparticle	Pt(IV)/CpG	[43]
Macrophage regulation	DSPE-PEG_2000_	Carrier	Nanoparticle	BLZ-945/selumetinib	[170]
	β-CD/lysine	Carrier	Nanoparticle	Resiquimod	[172]
	M2pep	Responsive domain	Nanorod	M2pep/Au	[176]
T lymphocyte activation	PEG_2000_	Carrier	Nanoparticle	OVA/Bec1	[183]
	PLGLAG	Assembly domain	Nanoparticle	^D^PPA-1/NLG919	[186]
	Poly-l-lysine	Carrier	Nanoparticle	Au/VC/carnosic acid	[187]
MDSC depletion	Tadalafil/ICG/Fe^3+^	Inducer/carrier	Nanoparticle	Tadalafil/ICG/Fe^3+^	[199]

Despite that the successful cases of supramolecular biomaterials in promoting tumor immunotherapy have been demonstrated, there are still several challenges that need to be addressed to optimize and translate these therapeutic strategies into clinical applications. From the perspective of supramolecular agents, the current application mainly focuses on the research field of tumor cell killing and immune cell regulation. One challenge is the heterogeneity and complexity microenvironment molded by both tumor cells and stromal cells, which creates barriers to designing supramolecular agents that selectively target specific molecular interactions. Another challenge is the potential instability of supramolecular nanomaterials due to the highly dependent noncovalent bonding forces. The instability of supramolecular assemblies might cause off-target effects and immune-related adverse events, which can limit the efficacy and safety of supramolecular agents. Therefore, in order to ensure the effective delivery of bioactive drugs and antigens, it is necessary to develop novel self-assembly strategies for the purposes of improving the stability and bioactivity of supramolecular biomaterials. From the perspective of drug development, the clinical transformation of supramolecular nanomedicines is extremely difficult for their inherent properties, including the complicated fabrication process, difficulty in mass production, as well as the lack of quality control standards. In addition, there is a requirement for further preclinical and clinical studies to evaluate the efficacy and safety of supramolecular agents in tumor immunotherapy. It commonly includes the development of reliable preclinical models to accurately reflect the complexity and heterogeneity of TME, as well as the conduct of well-designed clinical trials to assess the efficacy and safety of supramolecular agents in patient populations. Collectively, the development of supramolecular biomaterials not only consists in the progress of biomaterials themselves, but also in the exploration of their further clinical application and evaluation. While challenges remain, with continued innovation and collaboration between researchers and clinicians, supramolecular biomaterials have the potential to make a significant impact on the field of cancer immunotherapy in the years to come.
